# Modified Media for Repeated In Vitro Cutting Cycles of *Cannabis sativa* Without the Use of Cytokinin

**DOI:** 10.3390/plants14071138

**Published:** 2025-04-06

**Authors:** Molly McKay, James E. Faust, Matthew Taylor, Jeffrey Adelberg

**Affiliations:** 1 Plant and Environmental Science Department, 171 Poole Agricultural Center, Clemson University, Clemson, SC 29630, USA; jfaust@clemson.edu (J.E.F.); jadlbrg@clemson.edu (J.A.); 2 Curio Wellness, P.O. Box 5470, Towson, MD 21285, USA; matt.taylor@curiowellness.com

**Keywords:** micropropagation, hedging, fed-batch, cannabis, tissue culture, cytokinin

## Abstract

In vitro hedging; combined with the fed-batch liquid media process is an innovative system that generates multiple sterile plants without the use of exogenous cytokinin. This combined process was demonstrated with *Cannabis sativa* (‘Cherry1’, ‘BaOx’, ‘T1’, ‘Peach’) grown in vessels of three different physical states—stationary agar (A); stationary Oasis^®^ infused with liquid (OILs); and agitated Oasis^®^ infused with liquid (OILa). Vessels were pre-selected as control or supplemented; where supplement vessels received 15 mL DKW liquid media each cycle harvest. The number of shoot tips harvested; shoot length; and dry shoot mass from repeated cutting cycles was recorded. In a single harvest; ‘BaOx’ and ‘Cherry 1’ produced one shoot per plant from the original 15 planted on all treatments. ‘Peach’ and ‘T1’ produced less shoots on average; but the most in OIL treatments. All shoots harvested were longer in OIL compared to A; regardless of genotype. Over multiple cycles; ‘Peach’ and ‘T1’ were unable to reliably produce shoots on a repeated schedule and were, therefore, eliminated from the experiment. By cycle 3; maximum number of plants were produced; regardless of supplementation (‘Cherry 1’; 30; ‘BaOx’; 22). Shoot length was above 10 mm (planting standard) for both genotypes until after the third cycle (10 weeks) where number and quality decreased (nodes and internodes easily discerned). By the end of the experiment; the only shoots that remained productive for over 16 weeks and multiple repeated harvest cycles were those in OIL treatments with supplements.

## 1. Introduction

*Cannabis sativa* is a multipurpose plant in both the agricultural and medicinal industries. With the legalization of industrial hemp in the 2018 Farm Bill (<0.3% delta-9 tetrahydrocannabinol THC) and relaxed regulation of cannabis for medical and/or recreational use in certain states of the United States, there is an increasing demand for large scale production. Current operations depend on stem cuttings from mother plants, to produce genetically uniform female plants. This method reliably produces vegetative clones, but “mother” stock require large amounts of space, labor, and maintenance. It has also been observed that over time these mother plants become less vigorous, and a source of bacterial, viral, and fungal disease that may be transmitted to the cuttings [1]. As an alternative, micropropagation techniques offer more sanitary approaches for facilitating mass production of high quality disease free plants, and genetic integrity. Currently, well-defined protocols and optimization procedures that lead to successful micropropagation of cannabis are lacking and require research [2].

Cannabis genotypes display genetic variation in their response to common micropropagation practices for several reasons including explant health, and response to PGR’s [3]. Some genotypes exhibit specific problematic phenotypic responses and issues; thus there is a need for methods that work on a large scale for multiple diverse backgrounds [3]. Most cannabis micropropagation studies treat these issues by varying the application of plant growth regulators, especially cytokinin, in a single batch process where a single plant is rooted, cut, and transferred once. Exogenous cytokinin can be applied to break apical dominance, but studies often neglect long-term plant health in culture and during acclimatization [4]. Studies that have used these PGR’s report excessive hydration of tissue (hyperhydricity), poor growth, and little multiplication with erratic success linked to specific genotypes [5].

In vitro hedging is an innovative modification of micropropagation where shoot tips are repeatedly harvested from a single rooted plant in culture over multiple cycles [6]. Hedging efficiently produces multiple flushes of sterile explants quickly, since the plant is already rooted. *Cannabis sativa* was shown to be suitable for rapid (3-week) harvest cycles without cytokinin [7] but quality was diminished after the third harvest when the agar media collapsed, assumedly due to water loss. When combined with a fed-batch liquid media process, hedging vessels have the potential to remain productive over a prolonged period.

The fed-batch process involves supplementation of liquid media to vessels during the time of harvest. Over an 18-month period, fed batch liquid during hedging *Pinus radiata* shoots had better shoot quality than plants transferred every month to fresh agar [6]. Switching from gelled mediums to liquid medium have shown better growth in *Hydrangea quercifolia* when plants in liquid culture produced larger plants, with in turn had better greenhouse growth ex vitro, because of the increased availability of water and solutes in vitro [8].

The presence of a well-developed root system is pivotal for efficient water and nutrient uptake [9]. Roots in Oasis^®^ IVE (Smithers—Oasis Company, Kent, OH, USA) have a greater hydraulic conductance and xylem with secondary protoxylem bundles more like field-grown plants, due to aeration of the root zone [10]. With exposure to oxygen in the rooting tissues, there is potential for higher root respiration, and furthermore better acclimatization ex vitro. For hedging systems with multiple harvests, it is important to maintain a healthy root system to provide new flushes of shoot tips and increase multiplication numbers. The Oasis^®^ IVE also has physical pore space available for the infusion of liquid media supplementation at the time of harvest, that gelling mediums do not possess. This process is a modification of a temporary immersion systems such as bioreactors, where roots are aerated, and highly desired in a mass propagation scale [11]. By aerating the roots, problems that typically arise from in vitro culture of *Cannabis sativa* can be minimized, such as hyperhydricity.

In this work, the multiple harvest hedging system was used with the fed-batch system in three different physical states (stationary agar, stationary Oasis^®^ infused with liquid media, agitated Oasis^®^ infused with liquid media) to observe the potential effects on shoot quality and quantity over five repeated cycles. Four genotypes of *Cannabis sativa* were tested in each of the three different physical states and media supplementation was added to replace what was lost between harvests for experimental vessels only. Observations were made on five repeated cycle harvests measuring the amount of shoot tips harvested, shoot length, discernable leaves, and dry shoot weight.

## 2. Results and Discussion

### 2.1. The First Culture Cycle: Batch Culture

**Figure 1:**
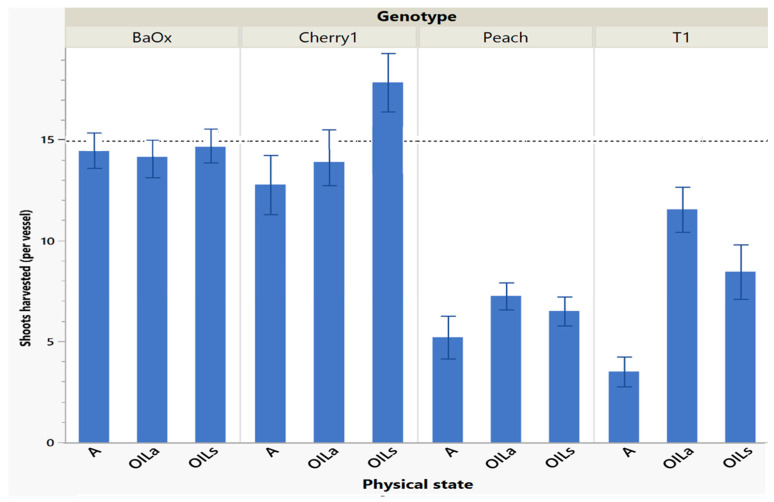
Number of shoots harvested in cycle 1 from RV750 vessels per genotype based on physical state agar (A), agitated Oasis^®^ infused with liquid (OIL_a_), or stationary Oasis^®^ infused with liquid (OIL_s_). Each standard error bar is 1 standard error from the mean, and the initial 15 plants per vessel is shown with a dotted reference line.

During the first cycle harvest (singular batch culture), the four genotypes differed in numbers of shoots harvested and length based on the physical state they were planted in. From the original 15 explants per vessel, ‘BaOx’ produced at least one shoot tip per plant regardless of physical state, while ‘Cherry 1’ produced less than 15 only in the A treatments (Figure 1). ‘T1’ and ‘Peach’ had fewer shoots than all genotypes, producing approximately only half the amount. ‘Cherry 1’ and ‘BaOx’, both had more shoots in OIL treatments compared to A. In ‘Peach’ and ‘T1’ genotypes, vessels that were placed on a rocker (OIL_a_) produced the most shoots. ‘T1’ produced approximately 12 shoots in OIL_a_, and ‘Peach’ produced 7 (Figure 1) In A treatments, ‘T1’ produced only 4 shoots, and ‘Peach’ produced 5. ‘Peach’ and ‘T1’ produced fewer shoots overall, compared to ‘BaOx’ and ‘Cherry 1’ (Figure 1). Most tissue culture protocols rely on a singular batch system transfer system with one cycle harvest and all four clones. While there was genetic uniformity within the four clones, which is not uncommon in cannabis micropropagation, overall all four genotypes in this work performed better in OIL_s_/OIL_a_ treatments.

**Figure 2:**
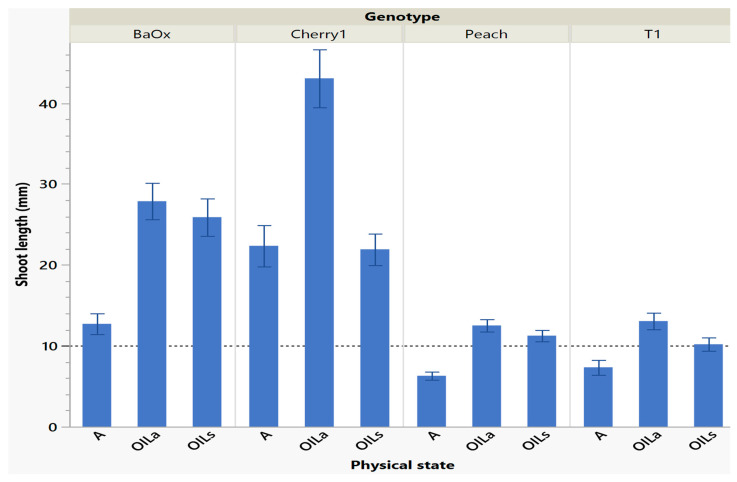
Length of a shoot harvested in cycle 1 from RV750 vessels per genotype based on physical state agar (A), agitated Oasis^®^ infused with liquid (OIL_a_), or stationary Oasis^®^ infused with liquid (OIL_S_). Each standard error bar is one standard error from the mean, and the desired harvest length of 10 mm is shown with a dotted reference line.

Shoots harvested from cycle 1 were the longest and had the most leaves, especially in OIL_s_/OIL_a_ treatments which was helpful for subculture (either in vitro multiplications or for subsequent ex vitro transfer for rooting). A quality plant that is appropriate for ex vitro acclimation must have functional roots and leaves [12]. Larger shoot tips often grow better because they have more leaves and discernable internodes (>10 mm). This desired planting length allows enough of the shoot base to be planted; with leaf tissue above media to maintain positive water potential which is crucial for a new microplant. Table 1 also suggests similarities between the number of shoots harvested, shoot length and number of leaves. The genotype ‘Cherry 1’ had so much extensive growth with (OIL_a_) that plants often curled under each other as they grew past the height of the RV750 vessel (90 mm) being approximately 44 mm during cycle 1 (Figure 2). ‘BaOx’ shoots were tallest in OIL_s_ treatments as well, approximately 26–28 mm (Figure 2). ‘Peach’ and ‘T1’ shoots in OIL treatments were also longer in cycle one and above the 10 mm planting when compared to A. ‘Peach’ was 12 mm in both OIL treatments, but only 6 mm in A. ‘T1’ was longest in OIL_a_ being 14 mm, followed by OIL_s_ being 10 mm and A being 8 mm (Figure 2). Similar results have been shown in other species that shoots grown in liquid culture have higher shoot lengths when compared to gelled mediums [8,13,14].

Over multiple cycles of hedging, ‘T1’ and ‘Peach’ failed with little internodal elongation that produced too few harvestable shoot tips, and some shoots of ‘Peach’ also had flowered in vitro. For these reasons, these two cultivars were removed from the multiple harvest experiment. The remainder of this manuscript follows multiple harvest cycles with the clones, ‘Cherry 1’ and ‘BaOx’.

### 2.2. Multiple Cycles of Hedging

**Figure 3:**
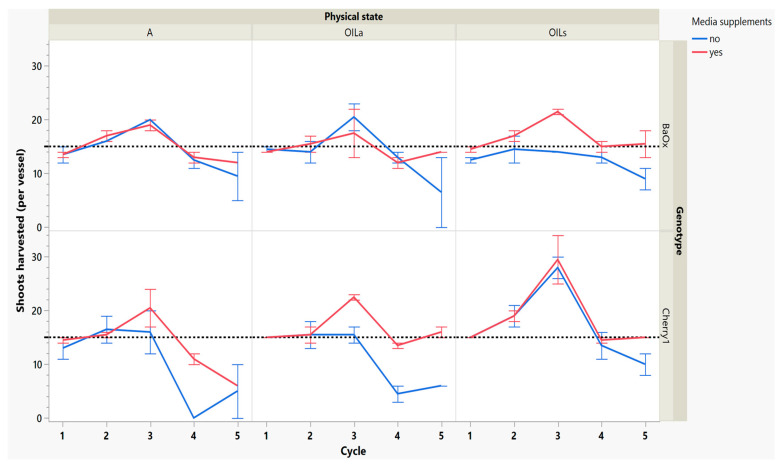
Number of shoots harvested from RV750 vessels per genotype over five repeated cycle harvests based on physical state agar (A), agitated Oasis^®^ infused with liquid (OIL_A_) stationary Oasis^®^ infused with liquid (OIL_s_). Media supplement of 15 mL indicated by red (yes) and blue (no media added). The initial 15 plants per vessel is shown with a dotted reference line.

After 3 weeks of regrowth, the second cycle harvest showed some increases in numbers of shoots harvested, especially in the genotype ‘Cherry 1’ where shoots harvested increased from 15 to 21 with OIL_s_ (Figure 3). The effectiveness of any micropropagation lab or optimization system depends on the proliferation rate and stability number of explants from a single donor plant [15]. Cannabis tends to produce a single shoot with a high degree of apical dominance, low levels of branching and low multiplication unless media recipes are altered to include PGR’s such as exogenous cytokinin [4]. The increase in multiplication rate of this work was not from exogenous cytokinin application, but rather the breaking of apical dominance during the first cycle harvest.

**Figure 4:**
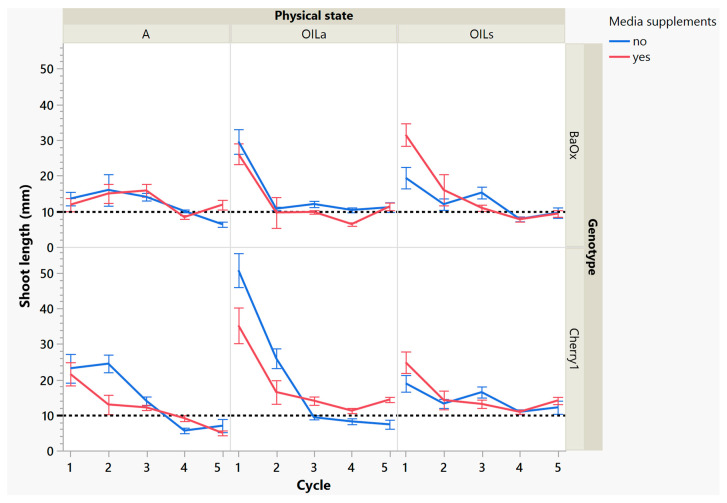
Length of a harvested shoot from RV750 vessels per genotype over five repeated cycle harvests based on physical state agar (A), agitated Oasis^®^ infused with liquid (OIL_a_) stationary Oasis^®^ infused with liquid (OIL_s_). Media supplement of 15 mL indicated by red (yes) and blue (no treatment added). The desired harvest length of 10 mm is shown with a dotted reference line.

**Figure 5:**
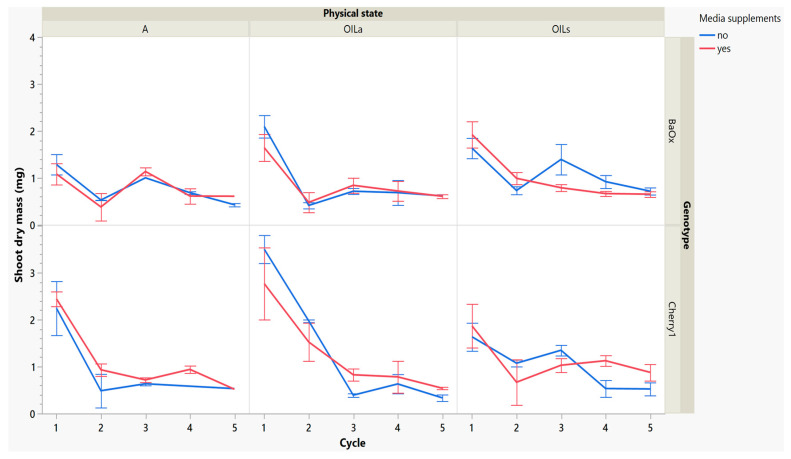
Dry mass of a shoot harvested from RV750 vessels per genotype over five repeated cycle harvests based on physical state agar (A), agitated Oasis^®^ infused with liquid (OIL_a_) stationary Oasis^®^ infused with liquid (OIL_s_). Media supplement of 15 mL indicated by red (yes) and blue (no treatment added).

While these treatments produced more shoots, the plants themselves were decreasing in leaf and shoot size, as well as dry mass (Figure 4 and Figure 5). During cycle 1, the average shoot length for the ‘Cherry 1’ genotype in OIL_a_ was 44 mm, while in A and OIL_s_ shoots varied between 20 and 25 mm (Figure 2 and Figure 4). By the second cycle, shoot length of ‘Cherry 1’ was similar in all physical states and the heavy flush of growth exhibited during the first cycle did not predicate any superior growth in subsequent culture cycles. For ‘BaOx’, shoot numbers started to increase in A treatments; regardless of media supplements, but OIL treatments; only started increasing with the additional media supplements. Shoot length also decreased rapidly in ‘BaOx’ from the average length of 26–28 mm in both OIL treatments (cycle 1) to 10–20 mm (Figure 2 and Figure 4). Shoots from ‘BaOx’ plants in A were not above 20 mm for the entire five cycles.

By cycle 3, both genotypes were producing maximum numbers of shoots harvested, and responded differently to supplemental media. ‘Cherry 1’ plants in A and OIL_a_ increased from approximately 15 to 20 shoots per vessel, but only with supplemental media (Figure 3). Without the supplements, the number of shoots remained around the initial 15 planted (Figure 3). ‘Cherry 1’ plants in OIL_s_ produced a maximum of 30 shoots per vessel regardless of supplements. Shoots were shorter in length than cycle 2, but did not decrease in size as drastically as they did during the period between cycle 1 to cycle 2 (Figure 4). The average shoot length during cycle 3 was approximately 18 mm in A for both genotypes (Figure 4). In OIL treatments, genotypes differed as ‘Cherry 1’ plants were averaging 15 mm with supplements in both OIL treatments, but without supplements OIL_a_ was 9 mm (Figure 5). For ‘BaOx’, supplementation did not increase the shoot length.

Dry mass also decreased over cycles, but ‘Cherry 1’ responded positively to media supplements, while ‘BaOx’ did not. In OIL_a_ and A, dry mass decreased from approximately 2–3 mg to approximately 1 mg before remaining constant. Dry mass continued to decrease without supplementations, but in the genotype ‘Cherry 1’ and OIL_s,_ shoot dry mass showed an increase (Figure 5). The genotype ‘BaOx’, in OIL_s_ and A treatments produced the maximum number of shoots. Plants grown in A produced the same numbers regardless of supplemental media, but OIL_s_ required supplementations to produce similar shoot numbers. The media supplementations did not help increase the shoot length, dry mass, or leaf number (data not given) in these vessels during this cycle.

**Figure 6:**
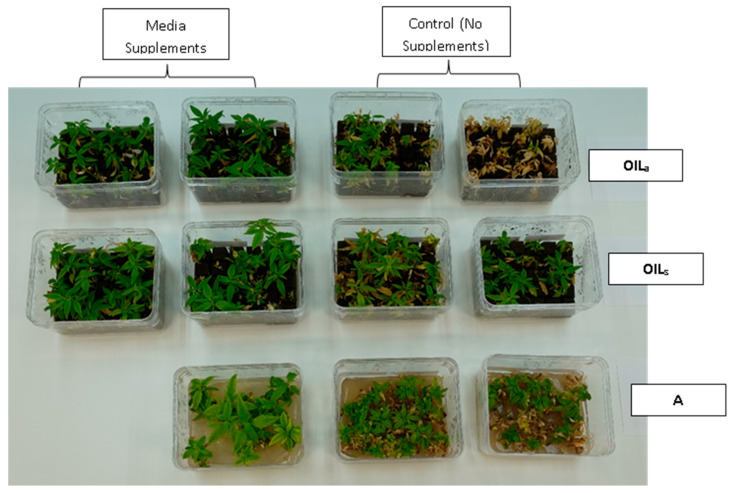
*Cannabis sativa* ‘BaOx’ in RV750 vessels after five multiple harvests (16 weeks after initial planting) in treatments with15 mL media supplements (**left**) versus no supplement (**right**) based on each physical state, Agar (A), agitated Oasis^®^ infused with liquid (OILa) stationary Oasis^®^ infused with liquid (OILs).

During the final cycles (4 and 5), plants in vessels with A treatments noticeably responded to a collapse in agar volume, and plant quality (leaves and internodes) deteriorated (Figure 6). Vessels containing ‘Cherry 1’ in A treatments without media supplements often faced mortality between the three week harvest period of cycle 4 and 5. By the final cycle, tissue harvest was below a minimal productivity threshold, (less than 7 shoot tips). Most vessels were eliminated from the experiment at the end of cycle 4, due to deterioration of plant health and quality or contamination. This was similar to the observations of previous research in cannabis species [7,16]. However, with the supplement of 15 mL at each harvest, plants in A were able to produce harvestable material into the fourth harvest, where 11 of the 15 shoots were harvested, still above the production level of 7 per vessel. Shoots from the genotype ‘BaOx’ averaged 10 mm, still above the planting standard. By cycle 5, even with media supplementation, the vessels with A were below planting size standard, and considered nonproductive (Figure 3 and Figure 6). In ‘Cherry 1’, shoot production from A vessels was low, and length was below planting standard of 10 mm. During the final cycle, the only vessels with actively producing shoots were in OIL_s_ vessels in both ‘Cherry 1’ and ‘BaOx’ (Figure 3 and Figure 6). Treatment A vessels were no longer productive because most cuttings coming from these vessels were stunted at approximately 4 mm (Figure 4). The only treatment vessels to perform over the entirety of five cycles and produce quality shoots at an appropriate planting length for both genotypes were OIL_s_ vessels.

## 3. Materials and Methods

### 3.1. Plant Material

*Cannabis sativa* ‘US Nursery Cherry 1’ (hereafter ‘Cherry 1’), ‘T1’, and ‘BaOx’ were aseptically subcultured from stage two stock cultures obtained from (USN Nurseries, Monroe, NC, USA) in non-vented Magenta GA -7 vessels (Magenta LLC, Lockport, IL, USA). The genotype ‘Peach’ was obtained from The Hemp Mine (Fair Play, SC, USA) and planted with a vented lid in a rectangular vessel with dimensions of 10 × 9 cm and capacity to hold 750 mL (RV750) (Smithers Oasis, Kent, OH, USA).

### 3.2. Experimental Design

Fifteen apical shoot tips or nodes approximately 1–2 cm in length were divided rectangular RV750 vessels with Oasis^®^ IVE infused liquid media (OIL) contained 120 mL of DKW medium (Driver and Kuniyuki, 1984, [17]) supplemented with 3% *w*/*v* sucrose, pH 6.2 and Oasis^®^ IVE (Smithers—Oasis Company, Kent, OH, USA). Agar vessels (A) contained an additional 7 g/L agar. Vessels were randomly planted and total initial volume of media was recorded with total vessel mass. Vessels were then wrapped with PVC sealing film (PhytoTech Labs, Lenexa, KS, USA), and labeled as control or experimental according to media supplementation. Experimental vessels received 15 mL DKW medium (Driver and Kuniyuki 1984, [17]), 3% *w*/*v* sucrose, pH 6.2 every harvest period, while control did not. Vessels were placed in treatment conditions with A and OIL_s_ vessels on one shelf, and a rocker platform at 5 oscillations per minute (Cole-Parmer, Vernon Hills, IL, USA) for the OIL_a_ treatment. All vessels were under similar light intensities (90–100 μmol·m^−2^·s^−1^) of 9 red:1 blue light emitting diodes (Hubbel NutriLED, Greenville, SC, USA) light for 16 h photoperiod. The first cycle harvest period for ‘Cherry 1’, ‘BaOx’ and ‘Peach’ was harvested at 4 weeks, and eight weeks for ‘T1’. The following harvest cycles for the rooted plantlets were 3 weeks (or 6 weeks for ‘T1’).

### 3.3. Data Collection

During harvest, vessels were unwrapped and weighed to measure water loss. Apical shoot tips were harvested, counted, and combined to determine fresh tissue mass per vessel. Each shoot harvested was measured for length (base of cut stem to apical shoot tip), and the number of leaves was counted. On assigned treatment vessels, media was supplemented and all were rewrapped with PVC sealing film (PhytoTech Labs, Lenexa, KS, USA) until next harvest period. Harvested shoot tips were enclosed in paper envelopes for 72 h in convection drying oven at 60 C for dry weight. This process was repeated for five, three week cycles. Vessels were terminated when the number of shoot tips harvested dropped below production standards (the minimum harvest number of seven shoots per vessel), biotic contaminations occurred, and one genotype was terminated when it flowered. Data were analyzed using JMP 16.1 (SAS Inst., Cary, NC, USA) with factors considered significant using a *p*-value of 0.01.

## 4. Conclusions

The fed batch process combined with in vitro hedging method is a useful process that improves production and optimization methods for some genotypes (‘BaOx’ and ‘Cherry 1’) of *Cannabis sativa*. The issues of genotypic variation still remain as we strive for common approaches for diverse genotypes, although in this work all four genotypes (Peach’ ‘T1’, ‘Cherry 1’ and ‘BaOx’) did produce more shoots in liquid media systems (OIL_s_/OIL_a_) compared to agar (A) in a traditional single harvest batch system. For multiple harvest schedules, ‘BaOx’ and ‘Cherry 1’ were productive for five repeated cycles but only with media supplements in the fed batch process. The only vessels to produce material over five cycles, with a quality shoot tip that could be planted above 10 mm ex vitro were vessels containing liquid media in Oasis^®^ (OIL_s_) that received cycle supplements. The decline in potential over five cycles was not a lack of nutrients or water deficiency as these were measured. Shoot length was decreasing and the internodal space became too short to cut in vitro. Increasing the shoot length and growth potential was addressed in a second experiment and chapter.

## Figures and Tables

**Table 1:** Summary ANOVA of shoots harvested, shoot length, leaves per shoot harvested, and dry mass per shoot for genotypes ‘Cherry 1’ and ‘BaOx’ in response to source factors cycle (C), genotype (G), physical state (P) and supplement of media (S) over five harvest cycle cuttings.

Source	Shoots Harvested	Shoot Length	Leaves per Shoot	Dry Mass per Shoot
Cycle (C)	<0.0001	<0.0001	<0.0001	<0.0001
C × C	<0.0001	<0.0001	<0.0001	<0.0001
Genotype (G)	0.6179	<0.0001	0.0797	0.0032
Physical state (P)	0.0042	<0.0001	0.0038	0.0580
Supplement (S)	0.0008	0.5124	0.6777	0.6365
C × G	0.1212	0.0038	0.0020	0.0003
C × P	0.3157	<0.0001	<0.0001	0.0043
C × S	0.0181	0.0847	0.0224	0.1822
G × P	0.0132	0.0079	0.2924	0.1058
G × S	0.4207	0.5373	0.8900	0.4798
P × S	0.7285	0.1322	0.5944	0.4944
Whole model R^2^	0.5600	0.3400	0.3800	0.7000

## Data Availability

The raw data supporting the conclusions of this article will be made available by the corresponding author on request.

## References

[1] Hesami M., Adamek K., Pepe M., Jones A.M.P. (2023). Effect of Explant Source on Phenotypic Changes of In Vitro Grown Cannabis Plantlets over Multiple Subcultures. Biology.

[2] Stephen C., Zayas V.A., Galic A., Bridgen M.P. (2023). Micropropagation of Hemp (*Cannabis sativa* L.). HortScience.

[3] Hesami M., Baiton A., Alizadeh M., Pepe M., Torkamaneh D., Jones A.M.P. (2021). Advances and Perspectives in Tissue Culture and Genetic Engineering of Cannabis. Int. J. Mol. Sci..

[4] Monthony A.S., Page S.R., Hesami M., Jones A.M.P. (2021). The Past, Present and Future of *Cannabis sativa* Tissue Culture. Plants.

[5] Baek S.-C., Jeon S.-Y., Choi Y.-J., Byun B.-H., Kim D.-H., Yu G.-R., Kim H., Lim D.-W. (2024). Establishment of an In Vitro Micropropagation System for *Cannabis sativa* ‘Cheungsam’. Horticulturae.

[6] Aitken-Christie J., Jones C. (1987). Towards automation: Radiata pine shoot hedges in vitro. Plant Cell Tiss. Org. Cult..

[7] Murphy R., Adelberg J. (2021). Physical factors increased quantity and quality of micropropagated shoots of *Cannabis sativa* L. in a repeated harvest system with ex vitro rooting. In Vitro Cell. Dev. Biol.-Plant.

[8] Adelberg J., Naylor-Adelberg J., Rapaka V. (2015). A novel rooting matrix and vessel system resulted in larger plants and faster growth during greenhouse acclimatization of *Hydrangea quercifolia* ‘Sikes Dwarf’. Propag. Ornam. Plants.

[9] Shi X., Collado C.E., Hernández R. (2024). Improve *Cannabis sativa* micropropagation through increasing air change rate in photoautotrophic and traditional tissue culture. Sci. Hortic..

[10] Adelberg J., Aitken J. (2023). Novel approaches to micropropagation, rooting and acclimatization. ActaHortic.

[11] Ioannidis K., Tomprou I., Mitsis V. (2022). An Alternative In Vitro Propagation Protocol of *Cannabis sativa* L. (Cannabaceae) Presenting Efficient Rooting, for Commercial Production. Plants.

[12] Economou A.S. (2011). From microcutting rooting to microplant establishment: Key points to consider for maximum success in woody plants. Acta Hortic.

[13] Adelberg J., Naylor-Adelberg J., Rapaka V. (2017). Phenolic foam rooting matrices allows faster transfer and more rapid growth of *Echinacea* plants in greenhouse. In Vitro Cell. Dev. Biol.-Plant.

[14] Ummugulsum E., Muhammet D. (2024). Enhancing in vitro micropropagation of *Alternanthera reineckii* Briq. using various light-emitting diodes, culture media and plant growth regulators. Kuwait J. Sci..

[15] Cavallaro V., Pellegrino A., Muleo R., Forgione I. (2022). Light and Plant Growth Regulators on In Vitro Proliferation. Plants.

[16] Kurtz L.E., Borbas L.N., Brand M.H., Lubell-Brand J.D. (2022). Ex Vitro Rooting of *Cannabis sativa* Microcuttings and Their Performance Compared to Retip and Stem Cuttings. HortScience.

[17] Driver J.A., Kuniyuki A.H. (1984). In Vitro Propagation of Paradox Walnut Rootstock. HortScience.

